# Applying Different Conditions in the OphthalMimic Device Using Polymeric and Hydrogel-Based Hybrid Membranes to Evaluate Gels and Nanostructured Ophthalmic Formulations

**DOI:** 10.3390/gels10080538

**Published:** 2024-08-20

**Authors:** Jonad L. A. Contarato, Geisa N. Barbalho, Marcilio Cunha-Filho, Guilherme M. Gelfuso, Tais Gratieri

**Affiliations:** Laboratory of Food, Drugs, and Cosmetics (LTMAC), University of Brasília, Brasília 72220-275, DF, Brazil; jonadlogan@gmail.com (J.L.A.C.); geisabarbalho@gmail.com (G.N.B.); marciliofarm@hotmail.com (M.C.-F.); gmgelfuso@unb.br (G.M.G.)

**Keywords:** 3D-printed device, drug delivery, ophthalmic hydrogels, polymeric membrane, retention time

## Abstract

The OphthalMimic is a 3D-printed device that simulates human ocular conditions with artificial lacrimal flow, cul-de-sac area, moving eyelid, and a surface to interact with ophthalmic formulations. All tests with such a device have used a continuous artificial tear flow rate of 1 mL/min for 5 min. Here, we implemented protocol variations regarding the application time and simulated tear flow to increase the test’s discrimination and achieve reliable performance results. The new protocols incorporated the previously evaluated 0.2% fluconazole formulations containing or not chitosan as a mucoadhesive component (PLX16CS10 and PLX16, respectively) and novel moxifloxacin 5% formulations, either in a conventional formulation and a microemulsion (CONTROL and NEMOX, respectively). The flow rate was reduced by 50%, and a pre-flow application period was also included to allow formulation interaction with the membrane. The OphthalMimic model was used with both polymeric and hydrogel-based hybrid membranes, including a simulated eyelid. Lowering the flow made it feasible to prolong the testing duration, enhancing device discrimination potential. The hydrogel membrane was adequate for testing nanostructure formulations. The OphthalMimic device demonstrated once again to be a versatile method for evaluating the performance of ophthalmic drug formulations with the potential of reducing the use of animals for experimentation.

## 1. Introduction

Prolonging the residence time of topical ophthalmic formulations is crucial for enhancing drug bioavailability and therapeutic efficacy [1,2]. Some eye drop formulations already include specific excipients to improve residence time, such as sustained-release agents, viscosity enhancers, and mucoadhesive substances [3,4]. However, they are often ineffective because approximately 95% of the administered dose is quickly drained through the tear ducts due to blinking. As a result, only a small fraction (5–10%) of the dose comes into contact with the ocular surface, leading to poor ocular bioavailability [5,6,7]. The bioavailability of ophthalmic topical formulations is also influenced by various factors such as the physicochemical properties of the formulation and the drug, the incorporation of polymers, and nanotechnology. Strategies like enhancing viscosity, employing in situ gel systems, utilizing mucoadhesive polymers, and implementing controlled release systems can notably enhance ocular bioavailability.

The current methods used to assess the effectiveness of these new formulations, mainly through in vivo and ex vivo testing, face challenges due to their expensive nature, variability in results and ethical concerns regarding animal experimentation [8,9]. Based on that, it is natural that over time, in vivo and ex vivo methods will be slowly reduced and replaced by in vitro methods, mainly those that have as principal characteristics high throughput, control, and predictability, and address many limitations of in vivo and ex vivo methods [10].

In vitro methodologies, such as the method using the OphthalMimic device, which utilizes simulated corneal models, are crucial in advancing ophthalmic drug development. To be truly effective, these models must demonstrate robustness to endure extended testing periods and be adaptable to assess a wide range of formulations [11]. The primary goal of employing in vitro techniques is to generate data that closely resemble the outcomes obtained from traditional in vivo studies, accurately simulating the conditions of topical ophthalmic administration. Establishing a direct correlation between in vitro and in vivo results (IVIVC) represents a significant milestone in drug development. This correlation is a valuable tool in improving the success rate of bioequivalence studies, streamlining the regulatory approval process for new formulations, and reducing the necessity for extensive animal experimentation. By offering a comprehensive insight into product quality attributes, the IVIVC supports a rational approach to drug development, facilitating informed decision-making across the product lifecycle, including potential post-approval adjustments [12].

Polymers are a class of biomaterials that have brought great advances in drug delivery, consequently contributing to progress in the health sector. These molecules, consisting of repetitive units called monomers, have several applications in drug delivery. They can be used for drug release (involving the drug, constituting a matrix that slowly releases the drug) and in tissue engineering, which has a wide range of applications such as skin, bone, cartilage, vascular, cardiac, cardiovascular, meniscus, human prostate tissues, artificial cornea, and others [13,14]. In the field of ophthalmology research, polyvinyl alcohol (PVA) has been extensively employed for the development of nanoparticles within PVA hydrogels, controlled-release PVA lenses, films, and nanofibers, owing to its biocompatible characteristics and capacity to create hydrogels [15,16,17]. In these senses, polyvinyl alcohol (PVA) is also recognized for generating mechanical strength membranes [18]. However, the hydrophilic nature of such membranes may compromise the resistance needed for more extended test periods exposed to simulated tear flow [19,20].

To most faithfully replicate the physiological application of topical ophthalmic formulations in vitro, our group has recently evaluated two types of entirely artificial membranes to simulate the cornea surface on the OphthalMimic device: polymeric membranes [11] and hydrogel-based hybrid membranes [21]. However, only a single condition with a continuous flow rate was tested when both membranes were applied, conducting a single test at a time.

Therefore, adjusting the device protocols to the membrane’s capabilities seems advisable to enhance testing efficiency and discriminatory potential. In this way, we describe the evaluation of different alternative protocol approaches for both polymeric and hydrogel-based membranes, namely the application of a pre-static condition as an alternative to continuous flow and the influence of a reduced flow rate, which were analyzed based on the performance of different types of formulations: conventional, mucoadhesive, and nanostructured. Owing to an upgrade in an OphthalMimic model, which now includes six test plates in sequence, it was also possible to assess the scaling up of the washing process. This enhancement has the potential to increase speed and practicality during formulation evaluation processes.

## 2. Results and Discussion

**Figure 1:**
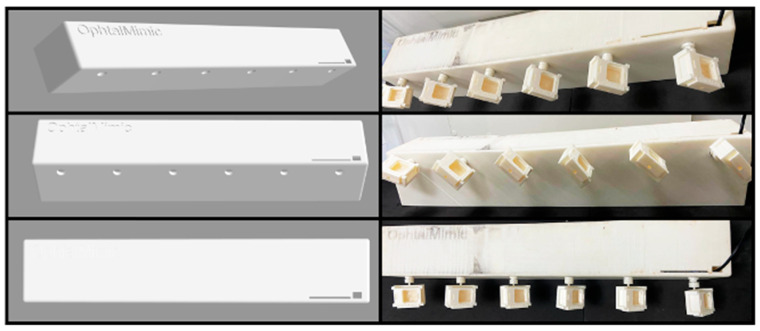
Scalable 3D-printed OphthalMimic model with 6 synchronized motors for connecting the tubes that provide simulated tear flow assembled before analyzing the formulations. On the left is the 3D design of the model, and on the right, the model is viewed from different angles.

The scalability of the OphthalMimic model for industrial processes is crucial for optimizing evaluation efficiency and agility and minimizing operational expenses. The model was meticulously designed with six synchronized motors to achieve this objective, facilitating the simultaneous replication of multiple samples (Figure 1). This novel approach enables the seamless connection of peristaltic pump tubes to individual ophthalmic molds, resulting in a dedicated collector for each sample. As a result, the analysis of a single formulation can be accomplished in just ten minutes, a notable improvement compared to the sixty minutes needed for a standalone model. This substantial time-saving enhancement significantly enhances the capacity for comprehensive formulation testing within a standard workday. The OphthalMimic model exemplifies the successful combination of scalability and cost-effectiveness. Through the utilization of 3D-printed bases, the production cost of the device is notably decreased, enabling the mass production of membranes.

Additionally, the model’s reusability and washability emphasize its practicality and sustainability [21]. To further enhance efficiency, a scale-up process was utilized to analyze hydrogel membranes under a 1 + 9 condition. This strategic approach accelerates the process by six times, reducing the total analysis time for twelve samples from a lengthy one hundred and twenty minutes to a quick twenty minutes.

Previously published studies have investigated membranes composed of different ratios of components. Notably, a polymeric membrane containing gelatin (2.5% *w*/*v*), PVA (10% *w*/*v*), and a combination of PLX and mucin in a 2:1 (*w*/*w*) ratio demonstrated favorable mechanical characteristics. These included a significant swelling capacity of over 80% after 40 min of exposure to an aqueous medium, minimal stretching leading to rupture at 8.55 ± 1.38 mm, and a deformation rate of 28.51 ± 4.61%. These findings underscore the structural robustness of the membrane under mechanical stress, a common occurrence during testing on the OphthalMimic device [11]. As a result, such a polymeric membrane was chosen to conduct the following protocol variations regarding flow and time in the OphthalMimic device. The polymeric membrane is a single-layer dry membrane containing mucin to simulate the corneal surface [22,23]. On the other hand, the hydrogel is a three-layer swollen membrane constituted by a basal hydrophilic layer, an intermediate mucous layer containing the same mucin, and a top phospholipidic layer. The hydrogel membrane is an advanced membrane compared to polymeric membranes because its swollen characteristic is essential for ocular tests, resembling the high water levels of the human cornea, ranging between 72% and 85% [24].

Our previous findings demonstrated the suitability of these membranes for assessing formulations incorporating mucoadhesive components. Nonetheless, to enhance the system’s discriminative capacity for formulations exhibiting more subtle mucoadhesive characteristics, such as those incorporating nanostructured systems, we have introduced protocol modifications aimed at prolonging formulation–membrane contact duration. By extending this interaction time, we anticipate a more pronounced differentiation of mucoadhesive properties, thereby refining the evaluation process and enabling a more precise assessment of these complex formulations [25].

**Figure 2:**
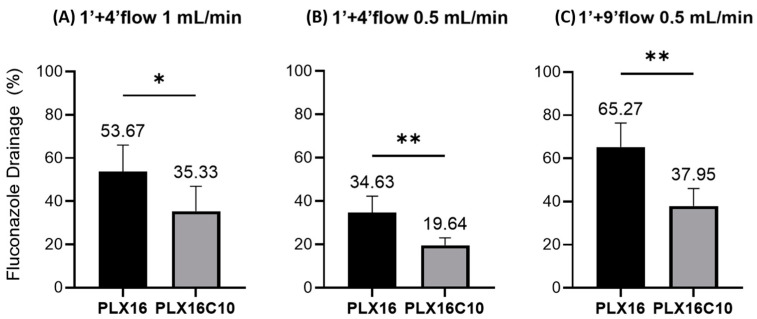
Percentage of fluconazole drained in the OphthalMimic device for the PLX16 and PLX16C10 formulations using a polymeric membrane (*n* = 6): (**A**) for 1 min of contact with the membrane without flow, followed by 4 min of continuous lacrimal flow at a 1 mL/min rate; (**B**) for 1 min of contact with the membrane without flow, followed by 4 min of lacrimal flow at a rate of 0.5 mL/min; (**C**) for 1 min of contact with the membrane without flow, followed by 9 min of lacrimal flow at a rate of 0.5 mL/min. Statistical analysis using a *t*-test confirmed significant differences between the formulations. * *p* < 0.05; ** *p* < 0.01.

The results presented in Figure 2A,B showed that the decrease in the simulated tear flow from 1.0 to 0.5% mL/min indeed improved the device’s discrimination power, providing a lower *p*-value for the latter. Previous studies have indicated that the membrane did not resist intact under a 9 min continuous flow of 1.0 mL/min [11]. Notably, the reduced flow rate also enabled a longer testing duration of 9 min, reducing stress on the membrane (Figure 2C).

**Figure 3:**
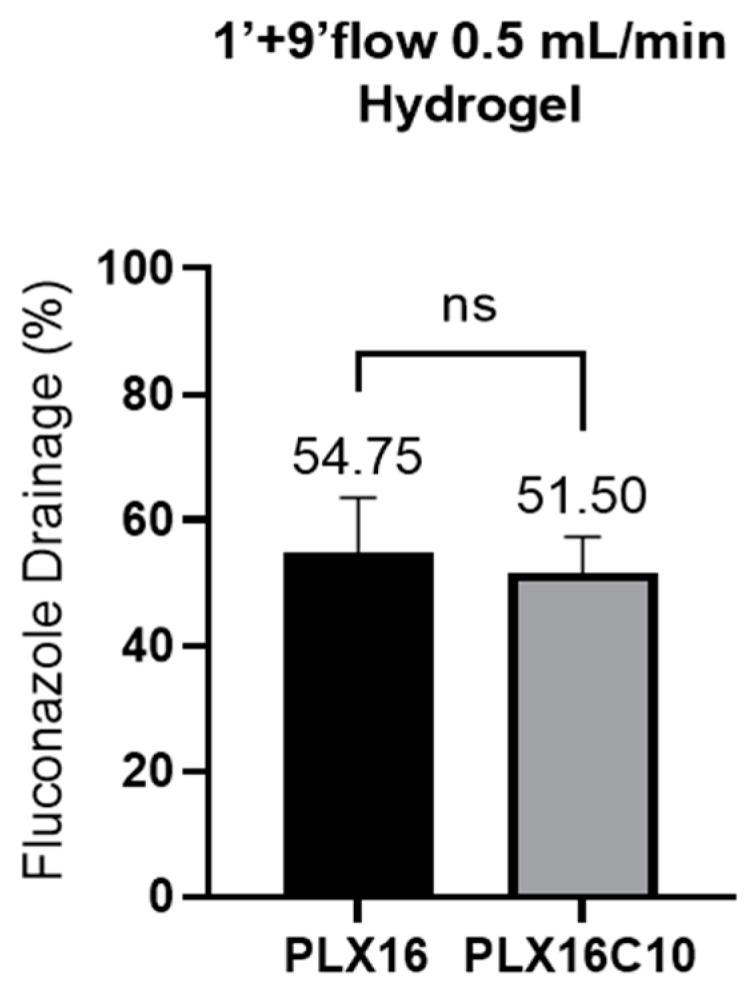
Percentage of Fluconazole drained in the OphthalMimic device using hydrogel membrane with artificial eyelid. Statistical analysis using a *t*-test showed no significant differences between the formulations (ns) (*n* = 6).

Prolonging the experimental duration resulted in a statistically equivalent outcome. Yet, the magnitude of difference in the absolute amounts drained was amplified, indicating an improved ability of the system to distinguish between formulations. The protocol of resting the formulation for 1 min without flow on the membrane surface and then applying a 0.5 mL/min simulated tear flow for 9 min was then applied to evaluate fluconazole residence time from PLX16 and PLX16C10 formulations using the hydrogel-based membrane (Figure 3).

As stated before, because the hydrogel membrane is more resistant than the polymeric one, the artificial eyelid could be incorporated into the device test, simulating the blinking movement. Surprisingly, the device would no longer differentiate between the two formulations (*p* > 0.05). On the one hand, the blinking movement improved the interaction between the control formulation (PLX16) and the base membrane, decreasing its drainage from 65.27 ± 11.06% to 54.75 ± 8.79%. Conversely, the same blinking movement promoted a higher drainage of the mucoadhesive formulation. Drainage increased from 37.95 ± 8.07% to 51.50 ± 6.16% (PLX16C10). The most plausible explanation for such an effect is the difference in formulations’ viscosity, as chitosan is known for its capacity to increase viscosity in gel formulations [26,27]. At room temperature, at which experiments were performed, i.e., at 32 °C, the apparent viscosities of PLX16 and PLX16C10 were, respectively, 0.372 and 146.370 Pa.s. Accordingly, in a previous experiment evaluating the hydrogel base membrane submitted to a continuous tear flow for 10 min under 1.0 mL/min simulated tear flow, also with eyelid movement, the drainage of the control formulation was comparable to that obtained with the current 1 + 9 min condition, PLX16 72.01 ± 3.57%, and the drainage from PLX16C10 formulation also increased to 64.73 ± 3.46%, but still the protocol was capable of differentiating both formulations (*p* = 0.0062) [21]. The present results indicate that adjusting the applied formulation amounts might be necessary for formulations with different viscosities.

Considering the promising landscape of new ophthalmic formulations, there is an increasing inclination toward the development of nanosystems for ocular administration [5,6,28,29]. These innovative systems offer a paradigm shift in ophthalmic drug delivery. Unlike traditional eye drops, which are frequently eliminated through blinking and tear production, nanosystems can provide prolonged drug delivery in smaller doses. This translates to a significant reduction in the frequency of application, improving patient compliance and adherence to treatment regimens. Moreover, nanovectors, owing to their distinctive size and characteristics, demonstrate a notable capability for engaging with corneal membranes [30]. This heightened interaction promotes enhanced drug absorption, resulting in enhanced therapeutic efficacy and potentially reducing the systemic side effects linked to traditional formulations. To optimize the efficacy of nano-based formulations, it is imperative to understand their residence time on the ocular surface. This duration is critical as it directly affects the bioavailability and therapeutic efficacy of the drug. Various physiological processes, including tear production, blinking, and drainage, play a role in determining this residence time. Improving our understanding of this crucial aspect can enhance the development and utilization of nanoformulations for ophthalmic applications. This enhanced knowledge will facilitate the development of more effective, safe, and adherence-friendly ocular treatments, leading to better patient outcomes.

Nanoemulsions possess the capacity to enhance ocular penetration and increase the bioavailability of drugs. Their nanometric size promotes a more efficient tissue interaction. In previous studies, we have conducted ex vivo investigations using the NEMOX nanoemulsion containing moxifloxacin to assess the drug’s corneal penetration. The drug’s high lipophilicity presents challenges in formulating conventional products containing it. Employing a nanoemulsion as a carrier for moxifloxacin and Poloxamer as a thickening and gelling agent appears to be a more advantageous strategy [31].

These experiments used simulated tear flow conditions to assess ocular penetration, employing an entire porcine ocular globe model with a custom-built donor compartment. Three formulations (NEMOX without PLX, NEMOX, and control containing 50% propylene glycol and 5% MOX) were applied to the corneal surface, and the system was maintained at 35 °C for two hours. The results indicated that the amount of drug that permeated the cornea was approximately 3.2 times higher in the nanoemulsion with PLX compared to the same nanoemulsion without PLX and the control containing 50% propylene glycol and moxifloxacin. This finding underscores the crucial function of PLX in facilitating drug delivery, possibly attributed to its capacity to modify the viscosity and surface characteristics of the nanoemulsion, thereby enhancing the interaction between the formulation and the corneal tissue.

Additionally, the nanoemulsion exhibited exceptional resistance to washing and drainage in static ex vivo experiments simulating artificial tear flow, with retention rates of MOX exceeding 80% after 2 h of exposure. The capacity to endure such circumstances not only enhances the drug’s retention on the ocular surface but also plays a role in achieving a more consistent and extended-release pattern, which is crucial for the efficacy of the therapy.

**Figure 4:**
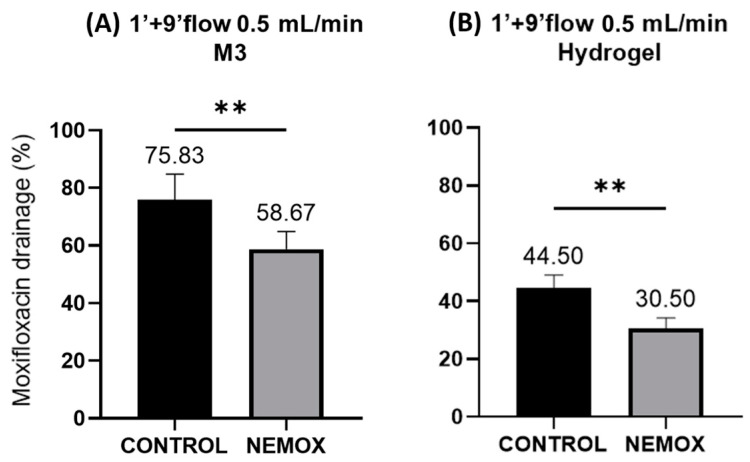
Percentage of moxifloxacin drained in the OphthalMimic device (*n* = 6): (**A**) moxifloxacin drainage using polymeric membrane without artificial eyelid; (**B**) moxifloxacin drainage using hydrogel membrane with artificial eyelid. Statistical analysis using a *t*-test confirmed significant differences between the formulations. ** *p* < 0.01.

In this way, we evaluated these same formulations in the OphthalMimic model. The comparison of this same protocol using the different membranes with different formulations but the same viscosity (one conventional—CONTROL and the other nanostructured—NEMOX with a viscosity of 2.036 Pa.s at 32 °C) shows that both polymeric and hydrogel-based membranes are capable of differentiating between the formulations (Figure 4B).

Polymeric and hydrogel-based membranes, when integrated with the Ophthalmic model, constitute a robust platform capable of significantly enhancing the efficiency and affordability of ophthalmic formulation development and preliminary testing. This innovative approach presents ample opportunities for further exploration, including evaluating diverse formulation types, such as lipophilic ophthalmic delivery systems encompassing micelles, liposomes, and nanoparticles. By expanding the application scope of this model, researchers can accelerate the preclinical assessment of novel therapeutic candidates, ultimately contributing to the development of more effective and accessible ophthalmic treatments.

The complexity of the tri-layered hydrogel has been significantly increased, along with its swelling capacity. Combined with the dynamic effect of eyelid movement, this augmentation has led to a more pronounced interaction at the base of the microsystem. Consequently, there has been a notable decrease in the volume of drained fluid compared to the static polymeric membrane. By integrating the simulation of the blinking reflex, the hydrogel-based method more accurately replicates the physiological conditions of the ocular surface, thus providing a more faithful representation of in vivo drug delivery. In contrast, the polymeric membrane-based approach, characterized by its extended exposure time and reduced tear flow rate, presents a unique experimental model. Although departing from the physiological norms, this approach may prove valuable in distinguishing formulation effects and could potentially enhance the feasibility of preclinical trials. The incorporation of this pre-static application technique could also contribute to a more comprehensive assessment of nanostructured drug delivery systems, such as microemulsions and liposomes. To fully elucidate the potential benefits of both approaches, further in-depth research is imperative.

## 3. Conclusions

In this study, protocol variations were implemented using the OphthalMimic device. The protocol that includes a 1 min static pre-application followed by 9 min of a lower continuous simulated tear flow provided enhanced device discrimination potential when evaluating different formulations. Formulation viscosity appeared to be a relevant factor when choosing the protocol, in which formulations with similar viscosity may be analyzed using the more complex hydrogel-based protocol, including a simulated eyelid movement. Still, the polymeric membrane base protocol with an extended time and lower simulated tear flow offers a more practical approach, enabling the fast screening of different formulations, enhancing research and development efficiency, and reducing costs and the number of animals for experimentation. This advantage is valuable for the development of new formulations and regulatory investigations.

## 4. Materials and Methods

### 4.1. Material

The necessary materials for this study were acquired from various suppliers. The scalable 3D-printed OphthalMimic model was built using 1.75 mm filaments of acrylonitrile butadiene styrene (ABS) supplied by 3DFila (Belo Horizonte, Brazil). Fluconazole, chitosan, PVA, mucin-type II, Poloxamer^®^ 407, 2-Hydroxy-4′-(2-hydroxyethyl)-2-methylpropiophenone, Irgacure^®^, and 2-methacryloyloxyethyl phosphorylcholine were purchased from Sigma-Aldrich (Steinheim, Germany). Gelatin type A was obtained from Vetec (Rio de Janeiro, Brazil). Sodium carbonate was acquired from Reagen (Colombo, Brazil), while sodium chloride and calcium chloride were purchased from Dinâmica (São Paulo, Brazil). Moxifloxacin was supplied by Eurofarma (São Paulo, Brazil). Ethyl oleate and Cremophor^®^ EL were obtained from Merck (Darmstadt, Germany), and Plurol^®^ Oleique was provided by Gattefossé (Lyon, France). High-performance liquid chromatography (HPLC)-grade acetonitrile and methanol were obtained from J.T. Baker (Phillipsburg, NJ, USA). Ultra-purified water from Merck Millipore (Illkirch-Graffenstaden, France) was used for all analyses.

### 4.2. Preparation of Polymeric and Hydrogel-Based Hybrid Membranes

Polymeric membranes were prepared with the casting technique, using a combination of PVA, gelatin, porcine mucin-type II (0.5% *w*/*v*), and Poloxamer^®^ 407 (1.0% *w*/*v*), as per the method described by [11]. Hydrogel-based hybrid membranes were synthesized using gelatin methacrylate and mucin methacrylate through photopolymerization, following the procedure outlined before [21].

### 4.3. Formulation Preparation and Drug Assay

For the analysis, two groups of different formulations were used: (i) a mucoadhesive formulation comprising 16% poloxamer, 0.2% fluconazole, and 1.0% chitosan (PLX16C10) compared to a conventional formulation comprising 16% poloxamer and 0.2% fluconazole (PLX16), and (ii) a microemulsion formulation comprising moxifloxacin 5% with poloxamer 5% (NEMOX), compared to a control 50% propylene glycol with 5% poloxamer containing 5% moxifloxacin (CONTROL). All samples were performed using a scalable OphthalMimic model.

Fluconazole (Log P: 0.5; water solubility: 1.39 mg/mL) 0.2% gels were prepared by solubilizing 16% of poloxamer in water or containing 1.0% of chitosan [32]. Fluconazole was analyzed using a chromatographic method at 210 nm with an injected volume of 20 μL in an isocratic system using acetonitrile, methanol, and water (15:5:80) as a mobile phase. The flow rate was 0.8 mL/min, and a C18 column (150 × 4.6 mm, 5 μm, Supelco^®^ Discovery BIO Wide-Pore obtained from Merck, Darmstadt, Germany) was used with the temperature set at 40 °C. The microemulsion moxifloxacin was prepared with a combination of surfactant and (Cremophor^®^ EL and Plurol^®^ Oleique, 4:1 *w*/*w*) dissolved in the oil phase, after adding the aqueous phase under continuous stirring at 1000 rpm at room temperature. Ending the preparation by adding 5% poloxamer under refrigeration [31]. A 50% propylene glycol aqueous control with 5% poloxamer was prepared by solubilizing 5% moxifloxacin. Moxifloxacin (log P: 2.9; water solubility: 0.168 mg/mL) was analyzed with a chromatographic method using a μporasil^®^ normal-phase column (10 μm, 125 Å, 3.9 mm × 300 mm, Cambridge, MA, USA). The mobile phase consisted of a mixture of solvent A (0.01 M phosphoric acid) and solvent B (methanol) in a 24:76 (*v*/*v*) ratio. The analysis was carried out at a flow rate of 0.8 mL/min with a sample injection volume of 25 µL. The column temperature was maintained at 40 °C, and UV detection was performed at 290 nm. The methods were evaluated using HPLC (model LC-20AD, Shimadzu, Kyoto, Japan).

### 4.4. Varying Test Conditions in OphthalMimic Device

**Figure 5:**
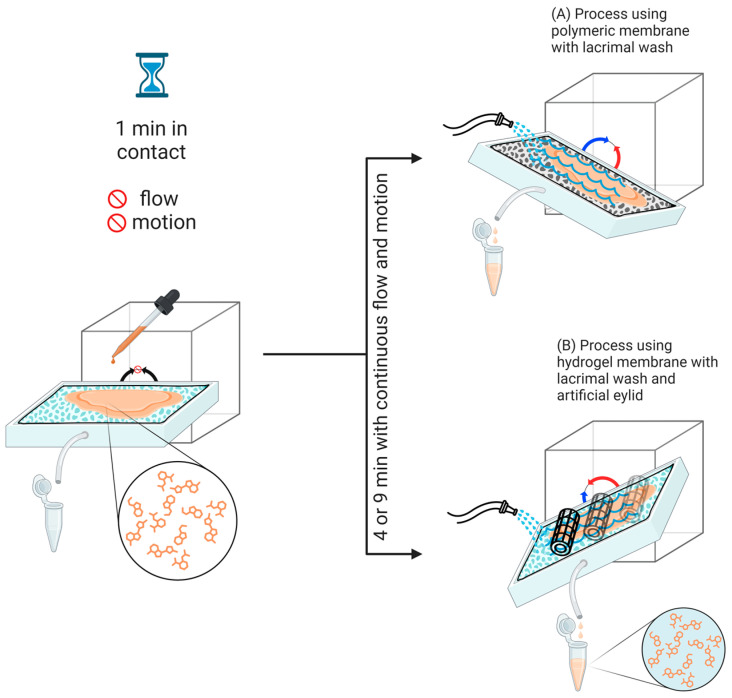
Schematic representation illustrating the washing process carried out by the OphthalMimic device. The process entails the application of a formulation above the membrane, a one-minute rest period, and the initiation of the washing process utilizing artificial tear flow for either 4 or 9 min, with or without the utilization of an artificial eyelid.

Resistance tests were conducted by applying 300 μL of each formulation. The simulated tear solution was prepared as previously described [33], which contained 0.2 g NaHCO_3_ (*w*/*w*), 0.67 g NaCl (*w*/*w*), 0.008 g CaCl_2_·2H_2_O (*w*/*w*), and H_2_O qs to reach a total of 100 g. Tests were conducted on two membrane types, namely a polymeric membrane and a hybrid hydrogel-based membrane, prepared according to Section 4.2. As the hydrogel membrane is more resistant than the polymeric one, it was only possible to incorporate the artificial eyelid into the hydrogel tests (Figure 5). In the device, the angular movement of the platform (ranging from 0° to 50°) causes the artificial eyelid to move at a frequency of 32 movements per minute, mimicking natural blinking. The fluid collected during the test was examined for analysis. Test conditions varied according to Table 1.

The lacrimal continuous flow was initiated 1 min after applying the formulation to enhance the interaction between the formulation and the membrane. The condition of 1 min without flow followed by 4 min of flow was tested with both regular and reduced flow rates. The same condition of a 1 min static pre-application followed by 9 min of continuous flow rate of 0.5 mL/min was evaluated on both membranes with fluconazole and moxifloxacin formulations. Moxifloxacin demonstrates higher lipophilicity in comparison to fluconazole, as evidenced by log *p* values of 2.9 and 0.4, respectively. The lipophilic characteristics of moxifloxacin may render it less suitable for incorporation into gel formulations. As a result, one of the most efficient approaches for the topical administration of this drug has been shown to be through a microemulsion, which enhances tissue penetration [31]. Thus, this nanostructured formulation containing a more lipophilic compound has proven to be a viable option for challenging the OphthalMimic device.

### 4.5. Statistical Analyses

The data were analyzed using GraphPad Prism^®^ Software, version 9.0.0 (San Diego, CA, USA), using the *t*-test variance method. The null hypothesis was confidently rejected at a significance level of *p* < 0.05.

## 5. Patents

Patent numbers: BR 10 2022 009997/BR 30 2023 005154 6 and BR 10 2024 014640 9.

## Figures and Tables

**Table 1:** Protocol variations tested on the OphthalMimic device, varying flow rate, membrane, and formulation analyzed.

Test Time	Flow Rate (mL/min)	Membrane	Formulation Tested	
1 min contact no flow + 4 min with flow	1.0	Polymeric	PLX16	PLX16C10
1 min contact no flow + 4 min with flow	0.5	Polymeric	PLX16	PLX16C10
1 min contact no flow + 9 min with flow	0.5	Polymeric	PLX16 CONTROL	PLX16C10 NEMOX
1 min contact no flow + 9 min with flow	0.5	Hydrogel	PLX16 CONTROL	PLX16C10 NEMOX

## Data Availability

The raw data supporting the conclusions of this article will be made available by the authors on request.
